# Targeted Osmotic Lysis of Highly Invasive Breast Carcinomas Using Pulsed Magnetic Field Stimulation of Voltage-Gated Sodium Channels and Pharmacological Blockade of Sodium Pumps

**DOI:** 10.3390/cancers12061420

**Published:** 2020-05-31

**Authors:** Dennis Paul, Paul Maggi, Fabio Del Piero, Steven D. Scahill, Kelly Jean Sherman, Samantha Edenfield, Harry J. Gould

**Affiliations:** 1 Department of Pharmacology and Experimental Therapeutics, Louisiana State University Health Sciences Center, New Orleans, LA 70112, USA; dpaul@lsuhsc.edu (D.P.); sscahi@lsuhsc.edu (S.D.S.); ksherm@lsuhsc.edu (K.J.S.); simbra@lsuhsc.edu (S.E.); 2 Department of Physics, Louisiana State University, Baton Rouge, LA 70808, USA.; paul.e.maggi@gmail.com; 3 Department of Pathobiological Sciences and Louisiana Animal Disease Diagnostic Laboratory (LADDL), Louisiana State University School of Veterinary Medicine, Baton Rouge, LA 70808, USA.; fdelpiero@lsu.edu; 4 Department of Neurology, Louisiana State University Health Sciences Center, New Orleans, LA 70112, USA

**Keywords:** targeted osmotic lysis, pulsed magnetic fields, advanced stage carcinoma, sodium channels, sodium pumps, Na^+^, K^+^-ATPase

## Abstract

Concurrent activation of voltage-gated sodium channels (VGSCs) and blockade of Na^+^ pumps causes a targeted osmotic lysis (TOL) of carcinomas that over-express the VGSCs. Unfortunately, electrical current bypasses tumors or tumor sections because of the variable resistance of the extracellular microenvironment. This study assesses pulsed magnetic fields (PMFs) as a potential source for activating VGSCs to initiate TOL in vitro and in vivo as PMFs are unaffected by nonconductive tissues. In vitro, PMFs (0–80 mT, 10 msec pulses, 15 pps for 10 min) combined with digoxin-lysed (500 nM) MDA-MB-231 breast cancer cells stimulus-dependently. Untreated, stimulation-only, and digoxin-only control cells did not lyse. MCF-10a normal breast cells were also unaffected. MDA-MB-231 cells did not lyse in a Na^+^-free buffer. In vivo, 30 min of PMF stimulation of MDA-MB-231 xenografts in J/Nu mice or 4T1 homografts in BALB/c mice, concurrently treated with 7 mg/kg digoxin reduced tumor size by 60–100%. Kidney, spleen, skin and muscle from these animals were unaffected. Stimulation-only and digoxin-only controls were similar to untreated tumors. BALB/C mice with 4T1 homografts survived significantly longer than mice in the three control groups. The data presented is evidence that the PMFs to activate VGSCs in TOL provide sufficient energy to lyse highly malignant cells in vitro and to reduce tumor growth of highly malignant grafts and improve host survival in vivo, thus supporting targeted osmotic lysis of cancer as a possible method for treating late-stage carcinomas without compromising noncancerous tissues.

## 1. Introduction

Metastatic carcinomas express high levels of voltage-gated sodium channels (VGSCs), a feature that imparts an increased ability to invade normal tissue and to metastasize [1,2,3]. This feature of epithelium-derived cancer cells provides the basis for proposed treatments using cytotoxic agents that target VGSCs with the goal to destroy or to block the function of these channels in an attempt to eliminate the cancer [3,4,5,6,7,8]. This approach has been able to slow tumor growth and metastasis by negatively modulating VGSC expression or function, but unfortunately, it does not kill the cancer cells [4,5,6,7,8,9,10,11,12,13,14,15].

By contrast, we have shown that the simultaneous administration of electrical or physiological stimulation of VGSCs and a pharmacological blockade of sodium, potassium-ATPase (Na^+^, K^+^-ATPase; sodium pumps) causes lysis of primary afferent neurons that over-express VGSCs without damaging neurons that express VGSCs at normal physiological levels [16]. We have thus proposed that, as in primary peripheral afferent neurons, the simultaneous activation of VGSCs and pharmacological blockade of Na^+^, K^+^-ATPase in cancer cells will result in an excess of intracellular sodium and increased osmotic pressure. In advanced-stage cancers that greatly over-express VGSCs, the rise in osmotic pressure is sufficient to cause osmotic lysis of the cancer cells, leaving unaffected cells that express VGSCs normally. When this “targeted osmotic lysis” (TOL) is administered to several lines of malignant cells in vitro using Na^+^, K^+^-ATPase-blocking cardiac glycosides in combination with electrical stimulation, we have been able to affect 100% lysis of the malignant cells. However, when TOL is similarly applied to ectopic xenografts of malignant breast cancer MDA-MB-231 cells in vivo, the response to treatment has been less complete [17].

**Figure 1:**
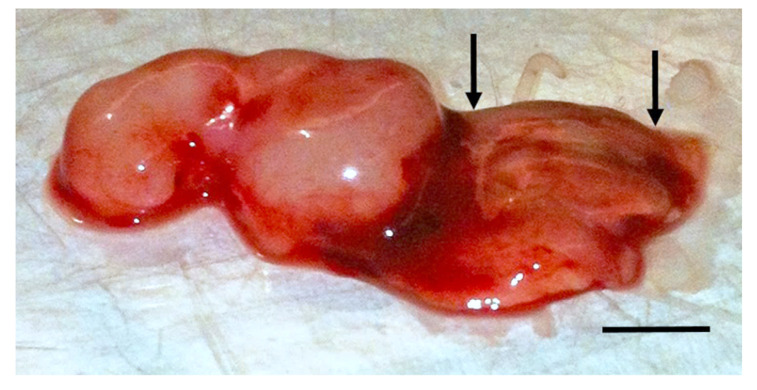
Evidence of tumor lysis following treatment with TOL using electric current stimulation. The photomicrograph depicts an ectopic, MDA-MB-231 xenograft that was removed 24 h after 3 treatments with TOL (10 mg/kg ouabain; 10 V, 1 msec pulses, 15 pps, DC electric current for 5 min). Due to the size and configuration of the stimulating electrodes, the stimulus had been delivered to only the portion of the tumor between the arrows. Note the obvious difference in gross appearance between the stimulated and unstimulated portions of the tumor. The stimulated area is necrotic. Calibration bar = 5 mm.

Two features of the TOL treatment as previously applied may contribute to limiting TOL efficacy and precluding optimum treatment in vivo. The first is the form of stimulation that we have used to activate VGSCs. Direct contact electrical stimulation introduces electrons into body tissues that then flow along the shortest path between the positively charged anodal source and the negatively charged cathode. The path of electron flow is determined by the degree of resistance imposed by the tissues that support the cancer cells. In biology, the tissues that comprise the extracellular tumor environment and the interstitial/extracellular tumor matrix provide a heterogeneous source of resistance that, as in electronics, can alter or inhibit the degree of stimulation that any given cell will receive [18,19,20]. Thus, as is evident in Figure 1, electrical current is not the ideal method for stimulating VGSCs especially in solid and deeply seeded tumors.

The second feature that may limit the efficacy of TOL in vivo is the ability to deliver sufficient cardiac glycoside to effectively block all of the sodium pumps in the cancer cells within a solid tumor. Variable vascularization, refractory drug transporters, and the composition of the extracellular tumor matrix consisting of collagen, elastin, fibronectin and laminin and the resulting intratumoral hydrostatic pressure, can limit the distribution of many chemotherapeutic agents by impeding blood supply, thus diminishing the efficacy of promising treatment options [20]. To this point, a number of agents designed to degrade the extracellular tumor matrix, such as losartan, an anti-hypertensive and anti-fibrotic drug that inhibits collagen I synthesis, and sildenafil, an agent that is used to treat erectile dysfunction by modulating the peripheral vascular tone and the distribution of blood supply through the inhibition of phosphodiesterase type 5 (PDE5), have been used to enhance drug penetration and the efficacy of chemotherapeutic agents in the treatment of cancer [19,21,22]. We therefore hypothesized that it might be possible to improve the efficacy of TOL for destroying advanced stage cancer cells by utilizing a more effective method for stimulating VGSC activation and by improving the delivery of the cardiac glycoside that is used to block sodium pumps.

It is well known that pulsed magnetic fields (PMFs) can be used to activate action potentials in excitable tissues [23,24,25,26]. Transcranial magnetic stimulation of motor cortex has been used clinically for the treatment of depression, movement disorders, post-traumatic stress disorder, migraine and chronic pain and has been shown to produce evoked potentials and movement in contralateral limbs [27]. As the delivery of magnetic field stimulation is much less influenced by tissue resistance than electric current stimulation [23,24,25,26], we hypothesized that PMFs would be a more efficient way to stimulate all cells in a body and improve lysis of invasive cancers. We therefore assessed the effect of TOL for treating highly malignant human (MDA-MB-231) and murine (4T1) breast cancer cells in vitro using PMF as the stimulus modality. PMF stimulation was also used in vivo to test the efficacy of TOL for treating ectopic xenografts and homografts of highly malignant murine breast cancer cells with and without losartan or sildenafil pretreatment to enhance the blood supply and delivery of cardiac glycoside.

## 2. Results

### 2.1. In Vitro TOL Treatment

**Figure 2:**
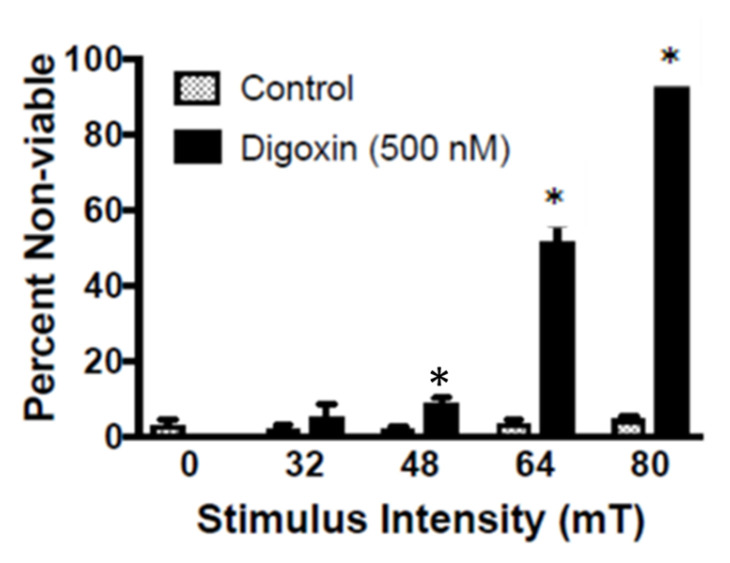
Stimulus-response curve for pulsed magnetic fields. MDA-MB-231 cells incubated in DMEM with or without 500 nM digoxin were treated with the indicated magnetic field stimulus intensity for 15 min. Subsequent cell counts revealed the stimulus dependence of TOL, with a maximum lysis rate of 95–100% cell death following TOL compared to 3–5% in controls. A two-way ANOVA revealed a main effect for treatment and a treatment X group interaction. * *p* < 0.05 by Tukey II post-hoc analysis.

MDA-MB-231 cells suspended in Dulbecco’s modified Eagle’s medium (DMEM) with 500 nM digoxin lysed within 15 min in a dose-dependent fashion when stimulated with an 80-mT PMF (Figure 2). Maximum lysis achieved was 95–100% at the 500 nM dose compared to the 3–5% that was observed in the three control groups (drug alone, stimulation alone, neither drug nor stimulation).

To assess the effect of PMF-induced TOL on normal cells, we used MCF-10a cells that minimally express VGSCs and do not metastasize. Using the 80 mT stimulus and 500 nM digoxin concentration, TOL had no effect on MCF-10a cells, with 96.5% of the cells appearing viable after treatment compared with 97.6% of the cells remaining viable after being treated with drug or stimulation alone (*p* > 0.8).

**Figure 3:**
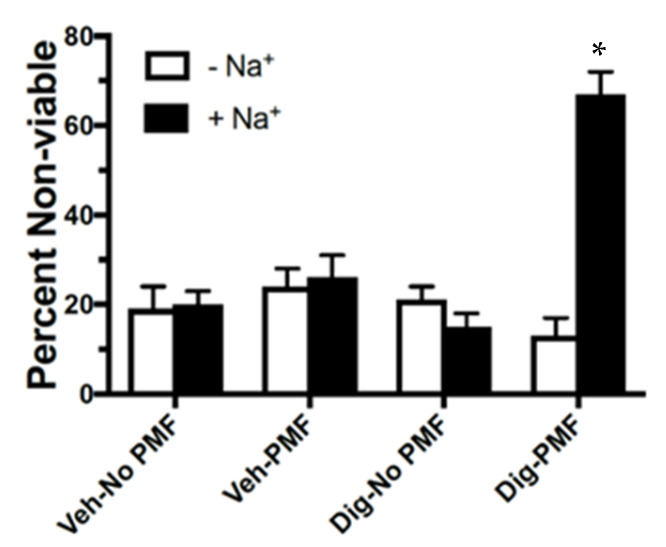
TOL dependence on Na^+^. The lysis of cells incubated in normal Ringer’s solution with 500 nM digoxin was 67% compared to the 15–22% lysis of cells in normal Ringer’s solution without digoxin when exposed to an 80 mT PMF. Counts of lysed cells incubated in Na^+^-free Ringer’s with digoxin and stimulated with PMF was comparable to controls. * *p* < 0.001 by planned orthogonal *t*-test.

To demonstrate that Na^+^ entry mediates this osmotic lysis, we assessed the effect of TOL in MDA-MB-231 cells that were incubated in Ringer’s solution with and without 500 nM digoxin and stimulated with 80 mT PMF. The 500 nM concentration is ½ log units below the minimally toxic concentration for digoxin alone. Sixty-seven percent of the cells that were suspended in Ringer’s solution with 500 nM digoxin and treated with PMF were lysed, compared to 15–22% lysis for the controls (*p* < *0*.001). MDA-MB-231 cells that were suspended in Na^+^-free Ringer’s media and similarly treated were indistinguishable from controls (Figure 3).

**Figure 4:**
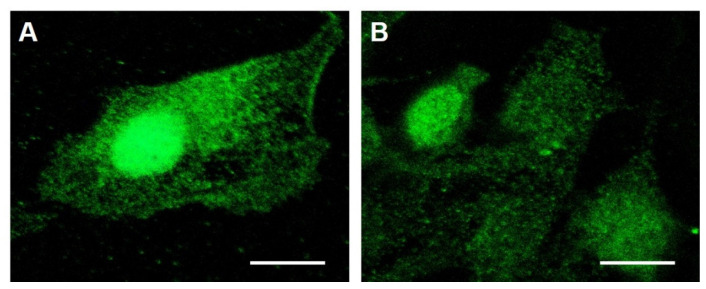
Sodium Channel Expression in MDA-MB-231 and 4T1 Breast Cancer Cells. Cultured cells were stained with a pan-specific anti-body for a conserved segment of the VGSC protein. Immunocytochemical imaging reveals labeling in cells of both MDA-MB-231 (**A**) and 4T1 (**B**) cell lines. The labeling depicted in the photomicrographs in Figure 4 was not observed in cells in which the anti-sodium channel antibody was pre-blocked with a 500-fold excess of the peptide antigen to which it was raised. The relative expression of VGSC proteins was confirmed quantitatively with flow cytometric analysis of the cell populations. Calibration bars = 15 µm).

To establish a basis for comparing the efficacy of TOL for treating triple-negative breast cancer in an in vivo immune competent model, VGSC expression was evaluated in 4T1 murine breast cancer cells using immunocytochemical staining and flow cytometry and compared with similarly treated MDA-MB-231 cells. Figure 4 depicts similar patterns of labeling of VGSCs in MDA-MB-231 and 4T1 cells using a pan-specific antibody of a conserved portion of the VGSC. Flow cytometry evaluation of the two cell populations revealed protein expression of VGSCs in 4T1 cells was similar to the expression in MDA-MB-231 cells (4.1 + 0.09 vs. 3.9 + 0.12–fold greater than autofluorescence). The MDA-MB-231 cells had been previously shown to express VGSC protein about 7.5-fold greater than “normal” MCF-10a cells [17]. Therefore, 4T1 cells also over-express these channels.

**Figure 5:**
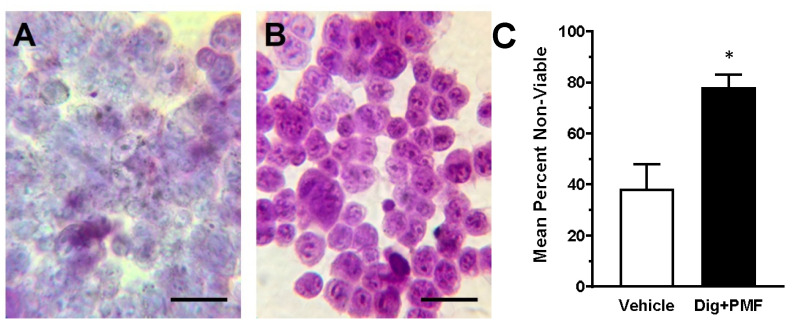
Targeted Osmotic Lysis of 4T1 Mouse Breast Cancer Cells. Cultured 4T1 cells in suspension were incubated for 15 min in DMEM + 500 nM digoxin or in DMEM alone, then stimulated with the pulsed magnetic field for 15 min. Assessment of viability was as with the previous experiment. (**A**): TOL-treated cells; (**B**): Vehicle-treated cells (calibration bars = 30 µm); (**C**): Cell counts comparing relative viability in samples that were treated with digoxin or vehicle and then exposed concurrently to an 80 mT PMF. *: *p* < 0.01 by *t*-test.

Using stimulus and dose parameters determined with MDA-MB-231 cells, 4T1 murine breast cancer cells were incubated in 500 nM digoxin and exposed to 10 min of an 80-mT PMF in order to compare the efficacy of TOL for treating triple-negative breast cancer cells that could establish a homograft tumor in an in vivo immune-competent (BALB/c) model. This TOL treatment significantly decreased the cells’ viability (Figure 5).

### 2.2. In Vivo TOL Treatment

**Figure 6:**
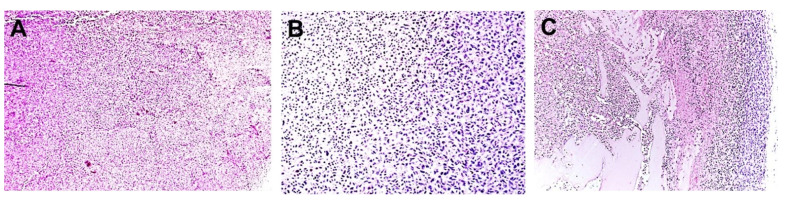
Tumor viability rating. The photomicrographs depict representative tissue sections selected by the blinded veterinary pathologist to illustrate the morphologic features of tissue samples taken from MDA-MB-231 tumors rated at 1 (**A**; no damage (there is no necrosis), ×10), 2.5 (**B**; moderate damage (necrosis with pyknotic cells), ×20) and 4 (**C**; significant damage (necrosis with cavitations), ×10). To date, we have been unable to reliably achieve complete tumor destruction, a Grade 5 response. The rating system was applied to the evaluation of samples from >200 mice.

**Figure 7:**
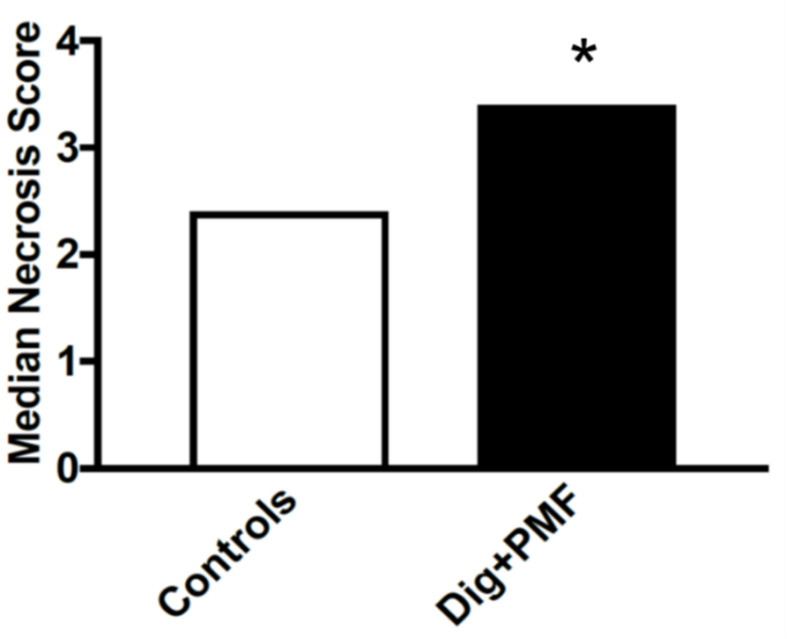
Relative viability of homografts. The average viability scores of TOL-treated xenografts compared to controls as determined by a veterinary pathologist who was blinded to the treatment provided are illustrated. Sections taken from 35 mice were used to determine the mean ratings of tumor viability for TOL-treated and control mice. Note that the TOL-treated tumors averaged 20–40% viability compared to 50–60% viability in control tumors. The three control groups were collapsed and compared to the tumors treated with both digoxin and PMF. * *Χ*^2^ *p* < 0.05. The necrosis observed in control can be largely attribute to damage seen during the natural history of a rapidly growing tumor.

**Figure 8:**
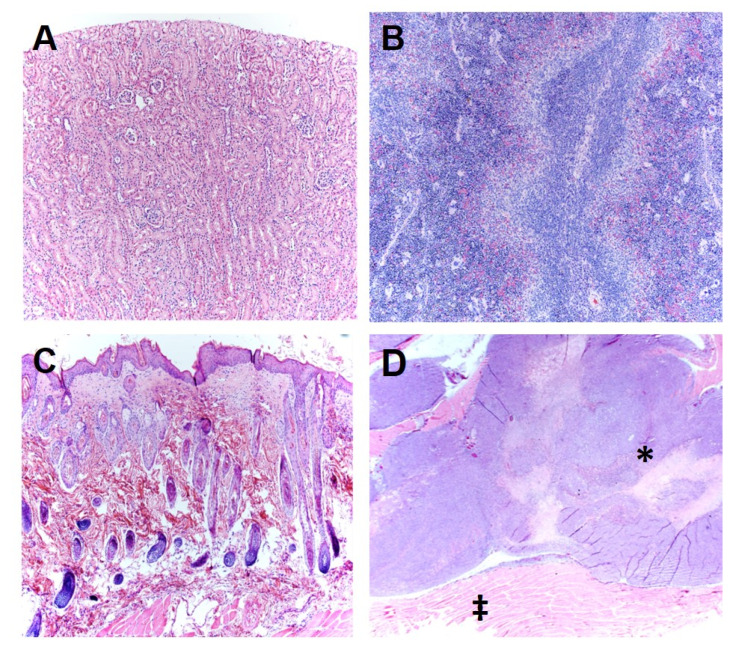
Morphology of representative tissues taken from the kidney (**A**; ×10), spleen (**B**; ×10), skin (**C**; ×10) and skeletal muscle (‡) adjacent to a homograft tumor (**D** (*); ×1.25) treated with TOL. The morphology of these tissues were determined to be normal, (although the skin has a nonsignificant lesion) indicating that it is unlikely that they were affected by treatment with TOL.

To determine the effect of TOL on tumor destruction, ectopic xenografts of MDA-MB-231 cells were established in immune-compromised J/Nu mice. The mice were injected with digoxin (7 mg/kg), a dose that is 30% less than the lethal dose in 1% of mice, then stimulated with PMF for 30 min on days 1, 3 and 5. The mice were euthanized 24 h after the last treatment and the tumors were removed, sectioned and stained with hematoxylin and eosin. Tumors from mice treated with digoxin and PMF (TOL) showed 80–100% tumor lysis (tumors were not found in 2 treated mice). Of the harvested tumors, tumor viability was assessed by a veterinary pathologist who was blinded to the treatments and rated tissue damage on a 1–5 scale: 1 indicated no damage, 2.5 indicated moderate damage and 5 indicated complete destruction of the tumor (Figure 6). A part of necrosis observed can be attributed to damage seen during the natural history of a rapidly growing tumor and on average is assumed to be similar across all xenografted tumors. Tumors treated with TOL averaged 20–40% viability (60–80% necrosis) compared to 50–60% viability (40–50% necrosis) in control tumors (Figure 7). Drug-only and stimulation-only controls did not differ from untreated controls and no damage was observed in normal tissues following TOL treatment (Figure 8).

**Figure 9:**
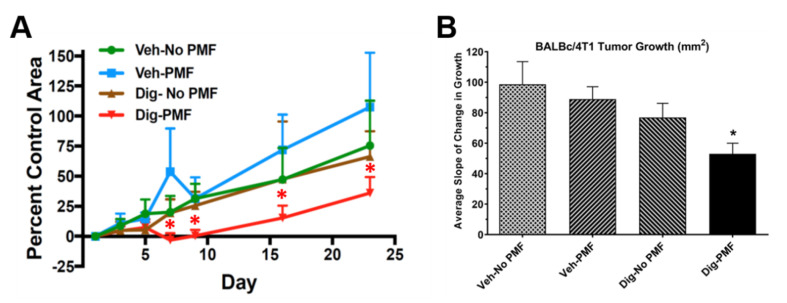
Growth of MDA-MB-231 xenografts (**A**) and 4T1 homografts (**B**) treated with TOL compared to the growth of xenografts that received drug or stimulation alone or vehicle. A. Groups of mice (*n* = 8) were treated as indicated. None of the mice that were treated with TOL (red curve) met NIH criteria for humane endpoint euthanasia. Three mice in the drug-only group (brown curve), 2 in the stim-only group (blue curve) and 2 in the vehicle-only group (green curve) met humane endpoint criteria and had to be sacrificed. B. The graph shows that the rate of growth of ectopic 4T1 homografts treated with TOL is significantly slower when compared to the growth of homografts that received drug or stimulation alone or vehicle. A one-way ANOVA revealed a main effect. * *p* < 0.05 compared to each of the controls by Tukey II post-hoc comparisons.

**Figure 10:**
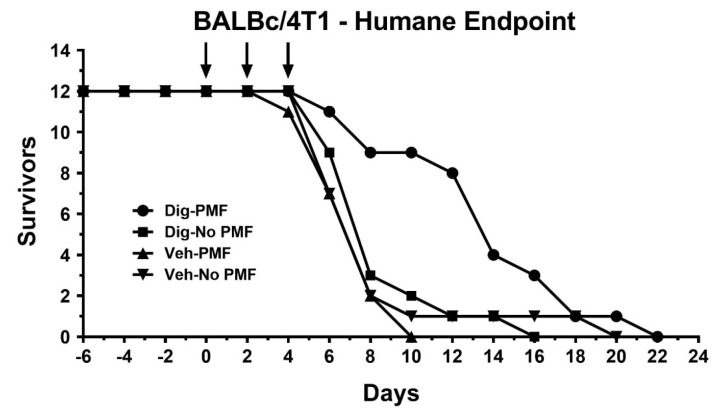
Post-treatment survival. Treating ectopic 4T1 homografts with TOL significantly increased survival when compared to the growth of homografts that received drug or stimulation alone or vehicle. *n* = 12/group.

**Figure 11:**
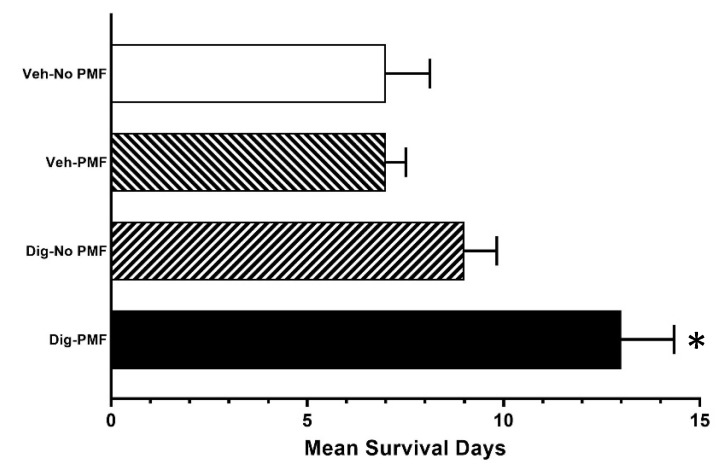
Time to 50% mortality (meeting NIH humane endpoint criteria for euthanasia). Treatment with TOL consistently extended the time to 50% survival for mice with 4T1 breast cancer homografts by approximately 1 week when compared to controls. One-way ANOVA revealed a main effect. * = *p* < 0.01 by Tukey II post-hoc comparisons.

To assess the effect of TOL using PMF on growth and survival, ectopic xenografts of either MDA-MB-231 cells or homografts of the highly malignant 4T1 murine breast cancer cells were established in immune-incompetent J/Nu nude or immune-competent BALB/c mice, respectively. The mice were treated as before and stimulated for 30 min on days 0, 2 and 4, then observed for 60 days. In both cases, survival was longer and tumor growth was slower in TOL-treated mice than in controls. Figure 9A shows the rate of tumor growth seen when treating MDA-MB-231 xenografts in nude mice. None of the TOL-treated mice met Nathional Institutes of Health – National Cancer Institute (NIH) criteria for humane endpoint euthanasia, but 3 mice in the group that received drug-only, 2 mice in the group that received only stimulation and 2 mice in the group that received the vehicle alone had to be sacrificed. Similarly, the rate of tumor growth seen when treating 4T1 xenografts in BALB/c mice is significantly slower (Figure 9B) and survival is significantly longer (Figure 10) when compared to controls. TOL treatment extended the time it took for 50% of the mice to reach NIH criteria for humane endpoint euthanasia by approximately 1 week (Figure 11).

In an attempt to improve the delivery of digoxin and enhance the efficacy of TOL, pretreatment with losartan, a drug that inhibits collagen I synthesis, or concurrent treatment with sildenafil, a modulator of peripheral vascular tone, was added to the treatment protocol. Tumor growth or host survival did not affect the TOL-treated or the control mice (data not shown).

## 3. Discussion

The results of the present study are in vitro and in vivo evidence that support our earlier observation that targeted osmotic lysis, the simultaneous stimulation of VGSCs and Na^+^, K^+^-ATPase blockade, is able to kill highly malignant MDA-MB-231 human and 4T1 mouse breast cancer cells while preserving similarly treated normal breast cells [17]. In the in vitro experiments, the lysis of the cancer cells was dependent upon the intensity of the PMF, with greater than 95% of the digoxin treated cancer cells lysing within 15 min, with as little as 80 mT PMF. As with our previous report, when Na^+^ was eliminated from the incubation buffer, lysis of the cancer cells was no different than controls. Thus, TOL is Na^+^-dependent.

We also demonstrated that the delivery of TOL using PMFs slows the growth of ectopic xenografts of both highly malignant human and homografts of murine breast cancer cells and increases survival of the hosts. Importantly, noncancerous organs in these animals were unaffected by the TOL treatment. Therefore, the cytotoxicity in tumors was not due to Rho/Rho kinase mediated apoptosis as has been seen with long-term treatment with cardiac glycosides [28]. The cells of normal tissues have only a fraction of the number of VGSCs compared to highly malignant cancer cells. Consequently, less Na^+^ and thus less water enters the cells, and they do not lyse [17].

The efficacy of treating xenografts with TOL using PMFs was comparable to treating with TOL using electrical current for slowing the growth of both the xenograft and homograft tumors, and increasing the survival of the hosts than when the tumors were treated with PMF alone, digoxin alone or vehicle, but the effect of TOL seemed to be less focused when PMFs were used. Moreover in neither case did TOL negatively affect noncancerous organs. Thus, this treatment is likely to be selective for cancerous tissue, sparing healthy tissue and reducing morbidity compared to current cancer treatments.

To assess whether our digoxin administration protocol was insufficient to perfuse tumors sufficiently, we used two treatments shown to increase chemotherapeutic pharmacokinetics [19,21,22]. Neither losartan, which degrades the extracellular tumor matrix [21], nor sildenafil, which increases vascular permeability [23], increased survival beyond our standard treatment protocol. Therefore, digoxin levels had achieved steady-state in all tissues of the body based on the drug’s pharmacokinetics, i.e., five doses separated by the digoxin t_1/2_.

PMF stimulation has the added benefits of providing a noncontact mode of stimulation and enabling the simultaneous targeting of all tumors in metastatic disease, even when collections of metastatic cells are too small to be imaged. This is because the neoplastic cells that over-express VGSC carry both the targeting feature and the mechanism necessary for producing a therapeutic effect.

In summary, we have shown in mouse models of breast cancer that PMF is a sufficient stimulus method to lyse carcinomas in a sodium-mediated TOL paradigm. Together, these results are strong evidence that PMF stimulation of VGSCs combined with blockade of Na^+^, K^+^-ATPase is a potentially efficacious treatment for late stage, highly malignant breast carcinomas.

## 4. Materials and Methods

### 4.1. Drugs

Digoxin was purchased from Sigma-Aldrich (St. Louis, MO, USA). For in vitro experiments, the drugs were diluted in the appropriate medium to a concentration twice that of the final concentration for that experiment. For in vivo experiments, digoxin was diluted in 10% dimethylsulfoxide (DMSO)/saline. Losartan and sildenafil were dissolved in saline.

### 4.2. Cell Culture

MDA-MB-231 human breast cancer cells were purchased from ATCC (Manassas, VA, USA) and cultured in Dulbecco’s modified eagle’s medium (DMEM; Gibco, Grand Island, NY, USA) supplemented with 10% fetal bovine serum (FBS; Gibco, Grand Island, NY, USA) and penicillin/streptomycin (pen/strep; Gibco, Grand Island, NY, USA). Insulin (10 µM; Sigma-Aldrich, St. Louis, MO, USA) was added 48 h before testing to assure sodium pump expression and activity. Mouse 4T1 breast cancer cells were also purchased from ATCC and cultured in RPMI-1640 media supplemented with FBS and pen/strep. MCF-10a normal breast epithelial cells were cultured in complete mammillary epithelial growth medium (MEGM; Lonza, Basel, Switzerland), which contains 10 µM insulin, supplemented with pen/strep.

### 4.3. Animals

All of our procedures were approved by the Louisiana State University Health Sciences Center Institutional Animal Care and Use Committee (IACUC), which is OLAW approved (Assurance #D16-00058), AAALAC accredited (#000037), and US Dept. of Agriculture certified (#72-R-0003). For MDA-MB-231 cell xenografts, 6-week-old, 15–20 g, female J/Nu mice were purchased from Jackson Laboratories (Bar Harbor, ME, USA) and housed in a specific pathogen-free, climate-controlled colony room with a 12/12 h light/dark cycle with free access to water and lab chow. To establish xenografts, approximately 4 million cells were injected in a 50% matrigel/saline suspension. Tumors formed to 0.7–1.2 cm diameter in 3–4 weeks.

For 4T1 homografts, 6-week-old, 15–20 g, female BALB/c mice were purchased from Jackson Laboratories and housed as with the J/Nu mice, but in a room with modified barrier controls. To establish homografts, approximately 500,000 cells were injected subcutaneously between the scapulae in a saline suspension. Tumors formed to 0.7 to 1.2 cm in 7 days. Treatment day 1 was initiated when average tumor size attained 5–10 mm in length as measured with a digital caliper by at least 2 investigators, one of which was not blinded to the treatment groups.

### 4.4. Flow Cytometry

MDA-MB-231 and 4T1 cells were dissociated using Cellstripper (Corning Life Science, Corning, NY, USA) centrifuged, decanted and resuspended in RPMI. 4% paraformaldehyde was added for 10 min. The cells were then centrifuged, decanted and washed with saline. The cells were resuspended in 5% goat serum + 3% bovine serum albumin for 1 hr. After centrifugation and aspiration of the supernatant, the cells were resuspended in PBS and incubated overnight with a pan-specific VGSC antibody (Alomone Labs; Jerusalem, Israel) that was pre-conjugated to an Allophycocyanin (APC) fluorophore, excitation 594 and 633 nm/emission 660 nm. The cells were centrifuged and resuspended in PBS and analyzed using a FACSCanto II flow cytometer (BD Biosciences, San Jose, CA, USA).

### 4.5. Image Analysis

MDA-MB-231 and 4T1 cells were incubated in RPMI for 2 days on a 22 × 22 mm slide cover. Media was aspirated and the cells were fixed with 0.5% paraformaldehyde for 10 min, rinsed 3 times for 5 min with 1 × PBS and then blocked with 5% goat serum + 3% bovine serum albumin for 1 h. Cells were then incubated overnight with the pan-specific VGSC antibody (Alomone Labs), 1:200 dilution. After overnight incubation with the primary antibody, cells were washed 3 times for 5 min with 1 × PBS. Cells were then incubated at room temperature for 45 min with a goat-anti-rabbit 488 Alexa fluorophore secondary, 1:800 dilution. Cells were washed 3 times for 5 min with 1 × PBS. Cells were incubated 10 min with DRAQ5 as a nuclear counterstain, dilution 1:600. Cells were rinsed and set with ProLong Gold antifade mountant. Images were taken on a Leica DMi8 confocal microscope (Leica Microsystems, Wetzlar, Germany) at 40 × oil magnification. As a control for nonspecific fluorescence, the anti-sodium channel antibody was pre-blocked with a 500-fold excess of the peptide antigen to which it was raised.

### 4.6. In Vitro TOL Treatment

Before testing, cells were dissociated with Cellstripper and approximately 150,000 cells resuspended in each 1.5 mL microfuge tubes using DMEM with or without digoxin (Sigma-Aldrich). Tubes were placed in an 8” length, 2.5” inner diameter solenoid with 697 turns (500 ft.) of 12 ga copper wire that gives an expected peak field strength of 4.4 mT/A with an expected resistance of 0.825 Ω. Based on measurements performed by driving 0.76 A at 0.60 V, the measured peak field strength using a F.W. Bell (Bedford, MA, USA) Model 4048 Handheld Gauss/Tesla Meter was 3.99 mT/A. The calculated resistance was 0.79 Ω. For stimulus/response experiments, 1–5 VDC current from an AE Techron (Elkhart, IN, USA) 7224 DC-extended AC amplifier was pulsed at 25 Hz, with a 10 msec ramp and fall, controlled by a Tektronix (Beaverton, OR, USA) AFG 3021B waveform generator, which produced 35–80 mT magnetic pulses in the solenoid. Cells were stimulated for 10 min. For dose/response experiments, cells were resuspended in 500 nM digoxin and stimulated for 10 min at 80 mT. Following each experiment, cells were fixed with 0.5% paraformaldehyde in phosphate buffer, pH 7.6, then stained with Cresyl violet. An evaluator blind to the treatment counted live vs. dead cells in at least 10 fields and a total of more than 100 cells for each treatment. Cells with a centrally located nucleus were scored as viable. Cells that were pyknotic or had no nucleus were considered nonviable.

### 4.7. Sodium Dependency

For Na^+^ dependency studies, cells were resuspended in normal Ringer’s solution [125 mM NaCl, 5.0 mM KCl, 2.0 mM CaCl_2_, 1.0 mM MgSO_4_, 10.0 mM glucose, 10.0 mM HEPES ] or Na^+^-free Ringer’s [NaCl was replaced with 250 mM sucrose to maintain osmotic pressure] with or without 500 nM digoxin. Approximately 150,000 cells were distributed to each 1.5 mL microfuge tube. Tubes were treated with 0 or 80 mT PMF, then evaluated as with the stimulus-response curve.

### 4.8. In Vivo TOL Treatment

For in vivo validation, groups of J/Nu mice (*n* = 8) hosting MDA-MB-231 xenografts were injected subcutaneously between the scapulae, five times at 1 h intervals with 7 mg/kg digoxin or an equal volume of the 10% DMSO/saline vehicle to bring digoxin or vehicle levels to steady-state for testing. The mice were treated on day 1 or on days 1, 3 and 5. On each of these days, were exposed to the PMF for 15 min starting 30 min after the last injection. For pathological analysis, mice were sacrificed by cervical dislocation and fixed with 0.5% paraformaldehyde in phosphate buffer 24 h after the last treatment. Samples of tissue from the grafted tumors as well as representative samples of kidney, spleen, skin, and muscle tissues were harvested, embedded in paraffin, sectioned and stained with hematoxylin and eosin. The tissue sections were then evaluated with light microscopy by a veterinary pathologist who is board certified by the American College of Veterinary Pathologists and was blinded to the treatments provided. Because the ranking was not continuous, the effect of TOL on tumors was compared using a Χ^2^ test.

Groups of BALB/c mice with 4T1 homografts were injected with 7 mg/kg digoxin or the vehicle and tested as with the J/Nu mice.

### 4.9. Survival Studies

Post-treatment survival for MDA-MB-231-J/Nu and 4T1-BALB/c mice were evaluated using the same treatment parameters as above. The cross-sectional area of the xenografts were measured every third day for up to 33 days. Cross-sectional area was calculated by measuring the length and width of the tumors (d1 and d2, respectively) and calculating the assumed oval area using the formula: A = (d1/2) × (d2/2) × π) Mice were sacrificed when they met the NIH criteria for humane endpoint euthanasia of laboratory animals in cancer studies. In two experiments, one using MDA-MB-231 xenografts and another using 4T1 homografts, the tumor cross-sections were measured every other day and the mice were allowed to survive until humane endpoint criteria were met.

### 4.10. Enhancement of Drug Penetration

Ectopic homografts of 4T1 murine breast cancer cells were established in female BALB/c mice. For TOL, mice were injected with digoxin (7 mg/kg), then stimulated with PMF (80 mT at 25 Hz) for 30 min on days 1, 3 and 5 and compared with controls. Groups of TOL-treated or control mice received either losartan (20 mg/kg/d; Sigma-Aldrich) beginning 2 days before TOL or sildenafil (1 mg/kg; Sigma-Aldrich) 15 min prior to each digoxin dosing and were compared to groups of mice that did not receive supplementary drug. Tumor growth was measured every other day. Animals were euthanized when they met NIH criteria for humane endpoint euthanasia.

## 5. Conclusions

We have provided evidence that TOL can kill both highly malignant human (MDA-MB-231) and murine (4T1) breast cancer cells in vitro and can reduce growth of xenografts and increase the survival of mice in vivo when compared with controls while sparing normal cells and tissues. In addition, because of the highly conserved and essential nature of dynamic sodium channel–sodium pump relationship for intercellular communication and maintenance of intracellular homeostasis, TOL may be effective in treating a wide range of malignant carcinomas. We conclude that the novel, targeted osmotic lysis approach warrants further study as a potential option for treating advanced stage carcinomas.

## 6. Patents

A patent for the technology described in this manuscript entitled, Targeted Osmotic Lysis of Cancer Cells—File No. 11M01 (Serial No. 13/552,909) Paul DJ and Gould HJ III was allowed on 12/30/2014.
